# Initial experience in colorectal robotic surgery in a university hospital: the first 100 consecutive cases using Hugo™ RAS platform

**DOI:** 10.1007/s10151-026-03302-y

**Published:** 2026-05-05

**Authors:** P. Brandão, M. J. Alves, E. Silva, M. Sampaio, A. C. Silva, M. D. Santos

**Affiliations:** 1 Colorectal Surgery Unit, Surgery Department, Centro Hospitalar Universitário de Santo António, Unidade Local de Saúde de Santo António, Porto, Portugal; 2 Unit for Multidisciplinary Research in Biomedicine, School of Medicine and Biomedical Sciences, University of Porto, Porto, Portugal; 3 ITR, Laboratory for Integrative and Translational Research in Population Health, Porto, Portugal

**Keywords:** Robotic surgery, Colorectal surgery, Minimal invasive surgery, Hugo™ RAS, Medtronic^®^

## Abstract

**Background:**

The Hugo™ RAS platform (Medtronic^®^), featuring an open-console design and modular configuration, represents a novel alternative to established robotic systems. Limited large-scale series of colorectal procedures using this platform have been published. This study aimed to evaluate the feasibility, safety, and learning curve of implementing the Hugo™ RAS platform for colorectal surgery in a center without prior robotic experience.

**Methods:**

We retrospectively analyzed 100 consecutive adult patients (median age 68 years; 51% male) undergoing elective colorectal resection using Hugo™ RAS between April 2023 and December 2024. Surgical indications included malignancy (78%), benign neoplasia, and inflammatory disease. Primary outcomes included operative time, blood loss, conversion rate, oncologic adequacy, complications (Clavien–Dindo classification), and length of stay. Learning curves were assessed via CUSUM analysis.

**Results:**

Median operative time was 180 min (IQR 147.5–240.0), with blood loss of 50 mL (IQR 50–100). No conversions occurred. R0 resection was achieved in 93% of applicable cases, with median lymph node harvest of 20. Overall morbidity was 28%, including 5% major complications (Clavien–Dindo ≥ IIIb) and zero grade IV/V events. Median stay was 6 days. Male patients had significantly higher complication rates (39.2% vs 16.3%, *p* = 0.011). Comparing first versus last 50 cases, complications decreased from 34% to 22% (*p* = 0.181), while major complications remained stable. CUSUM analysis revealed stabilization after approximately 50 cases.

**Conclusions:**

The Hugo™ RAS platform enabled safe and effective colorectal surgery with zero conversions and oncologic outcomes meeting established benchmarks. The learning curve stabilized at 50 cases with progressive reduction in minor complications. These results support Hugo™ RAS as a valuable addition to minimally invasive colorectal surgery.

## Introduction

Robotic-assisted surgery has emerged as a transformative approach in colorectal surgery, offering enhanced precision, ergonomic benefits, and improved visualization compared to conventional laparoscopic and open techniques [1]. Since the advent of the da Vinci Surgical System (Intuitive Surgical^®^) in the early 2000s, robotic platforms have gained widespread adoption, with evidence supporting their utility in complex pelvic dissections, sphincter-preserving procedures, and reduced conversion rates to open surgery [2, 3]. However, high costs, limited accessibility, and platform monopolization have prompted the development of alternative robotic systems, such as the Hugo™ RAS (Medtronic^®^), designed to address these barriers through modularity, open-console architecture, and cost efficiency [4].

As early adopters of the Hugo™ RAS platform, our institution aims to contribute to the growing body of evidence evaluating its feasibility, safety, and clinical outcomes in colorectal surgery. This article presents our unit’s initial experience with the first 100 patients undergoing robotic-assisted colorectal procedures using the Hugo™ RAS system. By detailing technical considerations, perioperative outcomes, and lessons learned, this study seeks to inform surgical communities about the potential role of Hugo™ RAS in expanding access to robotic surgery while maintaining high standards of care [5].

## Methods

### Study design and population

This single-center, retrospective cohort study analyzed the first 100 consecutive patients undergoing robotic-assisted colorectal surgery with our institution’s Hugo™ RAS system (Medtronic^®^) between April 2023 and December 2024. Inclusion criteria encompassed patients aged ≥ 18 years undergoing elective colorectal procedures. Exclusion criteria included non-robotic approaches or incomplete clinical records. Ethical approval was obtained from the institutional review board, and informed consent was waived due to the study’s retrospective nature.

### Surgical team experience and learning strategy

Both colorectal surgeons performing the procedures had extensive prior experience in advanced laparoscopic colorectal surgery, each having completed more than 200 complex laparoscopic cases before the start of this series. Neither surgeon had any prior hands-on experience in robotic surgery with Hugo™ RAS or any other platform. From the beginning of our robotic program, their operative activity shifted almost exclusively to Hugo™ RAS procedures to concentrate surgical time on developing proficiency.

The implementation strategy followed a structured pedagogical approach, with the first 10 consecutive cases being right hemicolectomies to establish standardized workflows, docking protocols, and team coordination before progressing to pelvic procedures. This consecutive series included all elective colorectal procedures meeting standard indications for minimally invasive surgery without systematic exclusion of complex cases, though emergency procedures and extreme ASA risk cases were avoided during the initial familiarization phase.

### Surgical technique and robotic workflow

All procedures were performed with the Hugo™ RAS platform using a four-arm configuration, including a 12-mm optical trocar and three 8-mm robotic ports. Pneumoperitoneum was maintained at 12–15 mmHg. Surgical steps adhered to oncologic principles for malignant cases, including complete mesocolic excision and total mesorectal excision for rectal resections.

Initially, we followed the standard setup configurations provided by Medtronic for each specific procedure type. However, as we progressed through the learning curve, we identified opportunities to optimize our approach based on practical experience and observed limitations.

After approximately 15 cases, we transitioned to a standardized 2 + 2 arm configuration (butterfly-type) for all procedures, regardless of the surgical quadrant. This configuration proved more versatile and reduced setup and logistics complexity. For each procedure, we first defined the specific anatomical target (e.g., hepatic flexure for right colectomy, pelvis for rectal resection), then positioned the trocars accordingly using either a straight-line or semilunar configuration, depending on patient anatomy and target location.

Critical to avoiding external arm collisions, we maintained a minimum spacing of 8 cm between each trocar, which we found to be optimal after experiencing collisions with closer spacing in early cases. The angles and tilts of each arm were adapted to individual patient characteristics and body habitus, highlighting the flexibility of the Hugo™ RAS platform’s modular design. This standardized yet adaptable approach improved workflow efficiency and facilitated teaching and team familiarization, as the same basic setup principles applied across all procedure types while allowing patient-specific and procedure-specific adjustments.

Re-docking was planned for select multiquadrant procedures (e.g., total colectomy, total proctocolectomy) to optimize access. No unplanned re-docking occurred as a result of technical issues. In selected low anterior resections involving tall or broad patients, instrument reach during splenic flexure mobilization was optimized by temporarily moving the monopolar scissors to the fourth robotic arm port and operating with three robotic arms, avoiding re-docking while maintaining adequate exposure.

Advanced bipolar energy was used for hemostasis and dissection. An assistant 12-mm port allowed suction/irrigation, stapling, and selective clip application. Suction/irrigation workflow and smoke evacuation were standardized during the first cases to improve visibility and reduce operative pauses.

### Data collection and variables

Demographic, preoperative, intraoperative, and postoperative variables were extracted from electronic medical records. Complications were graded using the Clavien–Dindo classification. Pathologic outcomes included resection margin status, lymph node yield, and TNM staging (7th AJCC edition) for malignant cases.

### Statistical analysis

Data were analyzed with SPSS^®^ version 29.0. Categorical variables were described with frequencies and percentages. Continuous variables were described with medians and interquartile ranges. Associations for Clavien–Dindo complications were performed with chi-square or Fisher exact test for categorical variables and Mann–Whitney test for continuous variables. A *p* value < 0.05 was deemed statistically significant. Learning-curve dynamics were assessed via cumulative sum (CUSUM) analysis for both global operative times and procedure-specific subgroups. CUSUM charts were computed as the cumulative sum of case-wise deviations from the mean operative time (global and procedure-specific). In a sensitivity analysis using the median as the reference, qualitative patterns were unchanged.

## Results

### Patient characteristics

The study included 100 consecutive patients with nearly equal gender distribution (51.0% male, 49.0% female) and a median age of 68.0 years (IQR 59.0–78.0). Most patients were classified as ASA 2 (60.0%), followed by ASA 3 (38.0%) and ASA 1 (2.0%). The median BMI was 24.5 kg/m^2^ (IQR 22.0–27.2). Comorbidities were present in 77.0% of patients, while 21.0% were smokers, 47.0% had prior abdominal surgery, 9.0% were under anticoagulation, and 12.0% had received neoadjuvant therapy.

### Surgical procedures and outcomes

Right colectomy was the most common procedure (37.0%), followed by anterior rectal resection (26.0%) and sigmoidectomy (15.0%). Other procedures included left colectomy (6.0%), rectopexy (3.0%), abdominoperineal resection (3.0%), total colectomy (3.0%), ileocecal resection (3.0%), total proctectomy with ileoanal pouch (2.0%), and ileocolic resection (2.0%).

**Fig. 1:**
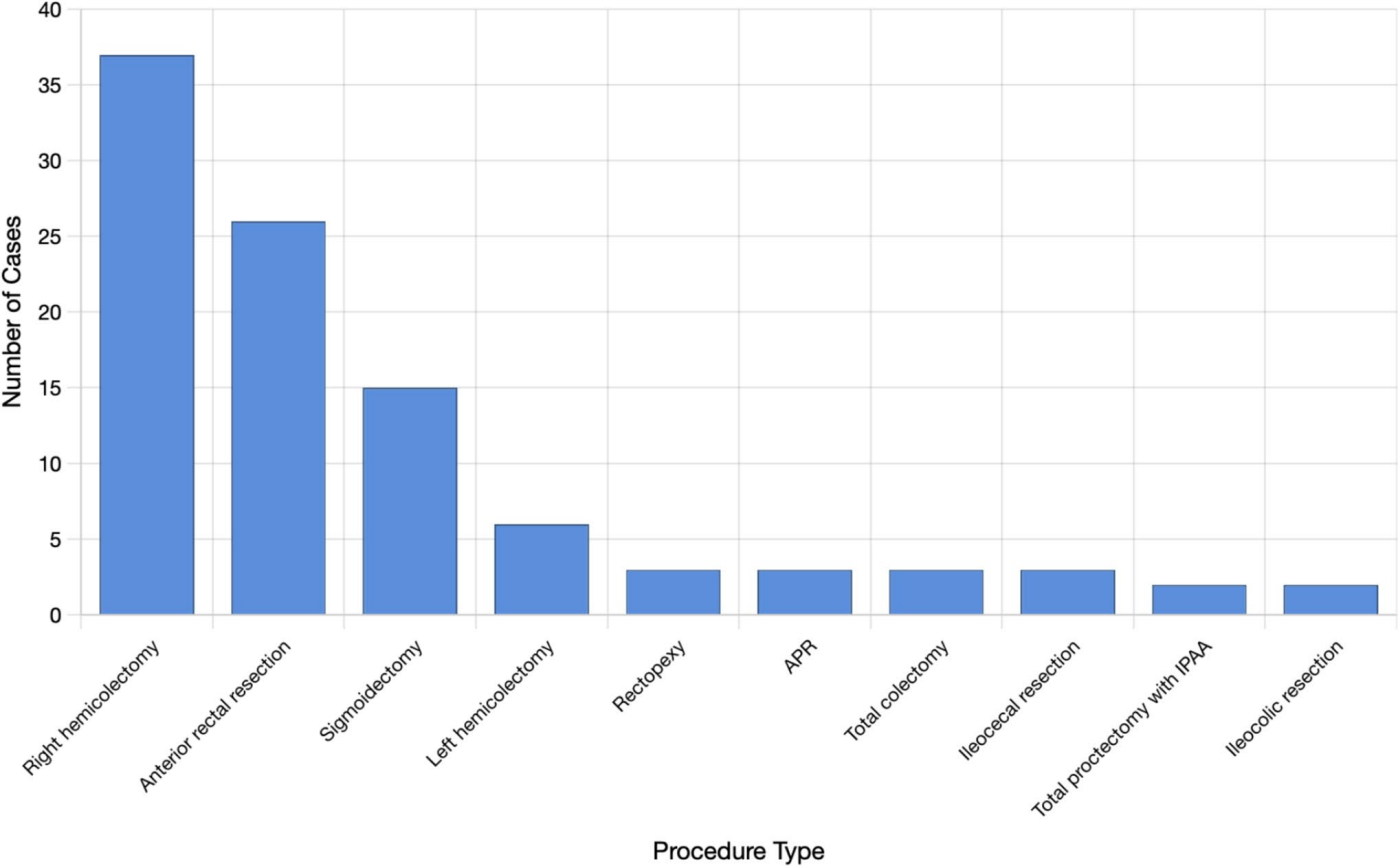
Distribution of surgical procedures performed with the Hugo™ RAS platform (*n* = 100). Right hemicolectomy was the most frequent procedure (37%), followed by anterior rectal resection (26%) and sigmoidectomy (15%). *APR* abdominoperineal resection, *IPAA* ileal pouch-anal anastomosis

The median operative time was 180.0 min (IQR 147.5–240.0), with estimated blood loss of 50.0 mL (IQR 50.0–100.0). No conversions to open surgery occurred. Stoma creation was performed in 20.0% of cases (Fig. 1). Resection margins were R0 in 93.0% of applicable cases. The median number of harvested lymph nodes was 20 (IQR 12–25). Malignant pathology was confirmed in 78.0% of patients.

The median length of hospital stay was 6.0 days (IQR 5.0–10.0). When stratified by pathology, median length of stay was 5 days for benign cases (*n* = 22) and 7 days for malignant cases (*n* = 78), *p* = 0.042.

### Complications

**Fig. 2:**
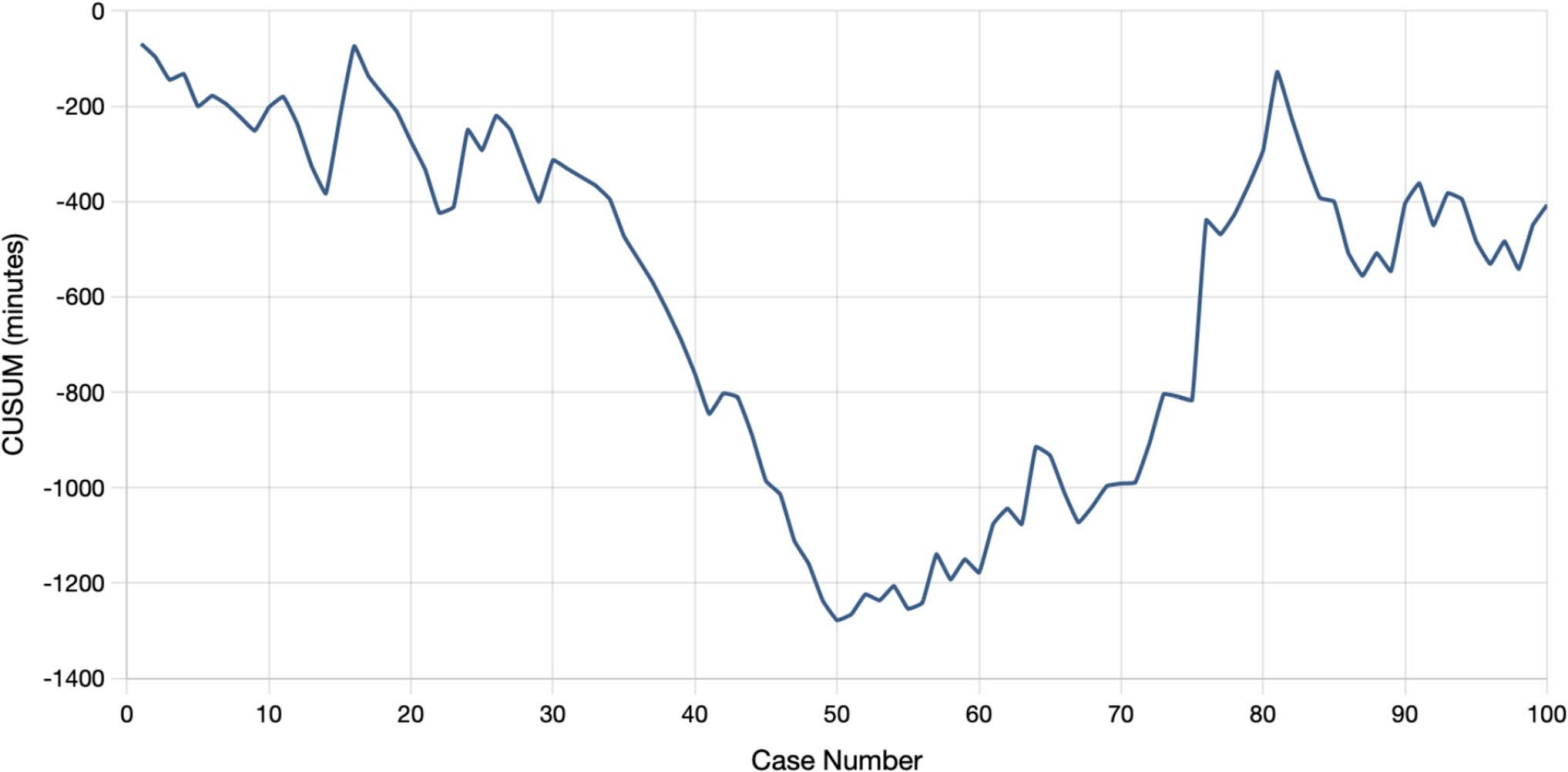
Global cumulative sum (CUSUM) analysis of operative times for all 100 consecutive cases. The *y*-axis represents cumulative deviation from the mean operative time (198 min, indicated by the horizontal dashed line at zero). The initial downward deviation (cases 1–10) reflects the deliberate strategy of beginning with right hemicolectomies, which had shorter operative times than the global mean. The curve stabilized after approximately 50 cases, oscillating around the baseline, indicating achievement of consistent operative performance

Global postoperative morbidity was 28.0%. According to the Clavien–Dindo classification, 14.0% had grade II complications, 4.0% had grade I, 4.0% had grade IIIa, and 5.0% had grade IIIb. No grade IV or V complications occurred. Re-intervention was required in 3.4% of patients (Fig. 2).

The most frequent complications were intra-abdominal abscess (8.0%) and urinary tract infection (6.0%). Other complications included metabolic ileus (3.0%), pneumonia (3.0%), and anastomotic dehiscence (3.0%).

Among analyzed factors, male gender was significantly associated with Clavien–Dindo complications (*p* = 0.011), with 39.2% of male patients experiencing complications compared to 16.3% of female patients. No significant associations were found for age ≥ 65 (*p* = 0.413), BMI ≥ 30 (*p* = 0.499), presence of comorbidities (*p* = 0.197), or neoadjuvant therapy (*p* = 0.176).

### Temporal analysis

Comparing the first 50 cases to the last 50 cases, overall complication rates decreased from 34% to 22% (*p* = 0.181). This improvement was primarily driven by reduction in minor complications (Clavien–Dindo grades I–II), while severe complication rates (≥ grade III) remained low and stable (8.0% vs 10.0%, *p* = 1.000).

### Procedure-specific outcomes

When stratified by procedure type, anterior rectal resection had the highest overall complication rate (34.6%), while sigmoidectomy and rectopexy recorded no severe complications. Left hemicolectomy showed the longest median length of stay (12.5 days) and highest rate of severe complications (16.7%), though the sample size was small (*n* = 6).

### Learning curve analysis

The global CUSUM analysis revealed a learning curve that stabilized after approximately 50 cases, with operative times consistently oscillating around the mean of 198 min (Table 1). The initial phase showed an expected downward deviation, reflecting the strategic choice to begin with right hemicolectomies, followed by progressive stabilization.

**Table 1:** Patient demographics and baseline characteristics (*n* = 100)

	Total (*n* = 100)
Gender	
Male	51 (51.0%)
Female	49 (49.0%)
Age (years)	68.0 (59.0–78.0)
ASA grade	
ASA 1	2 (2.0%)
ASA 2	60 (60.0%)
ASA 3	38 (38.0%)
BMI (kg/m^2^)	24.5 (22.0–27.2)
Comorbidities	77 (77.0%)
Smoking	21 (21.0%)
Prior abdominal surgery	47 (47.0%)
Anticoagulation	9 (9.0%)
Neoadjuvant therapy	12 (12.0%)
Procedure	
Right colectomy	37 (37.0%)
Anterior rectal resection	26 (26.0%)
Sigmoidectomy	15 (15.0%)
Left colectomy	6 (6.0%)
Rectopexy	3 (3.0%)
Abdominoperineal resection	3 (3.0%)
Total colectomy	3 (3.0%)
Ileocecal resection	3 (3.0%)
Proctectomy with ileoanal pouch	2 (2.0%)
Ileocolic resection	2 (2.0%)
Total operative time (minutes)	180.0 (147.5–240.0)
Estimated blood loss, mL	50.0 (50.0–100.0)
Conversion	0 (0.0%)
Tumor length (cm)	5.4 (1.3–9.5)
Resection margins	
R0	80 (93.0%)
R1	0 (0.0%)
NA	6 (7.0%)
Harvested nodes	20.0 (12.5)
Pathologic diagnosis (malignant)	78 (78.0%)
Length of stay (days)	6.0 (5.0–10.0)
Intraoperative complications	3 (3.0%)
Postoperative morbidity	28 (28.0%)
Complications according to Clavien–Dindo	
I	5 (4.0%)
II	14 (14.0%)
IIIa	4 (4.0%)
IIIB	5 (5.0%)
IVa	0 (0.0%)
V	0 (0.0%)

**Fig. 3:**
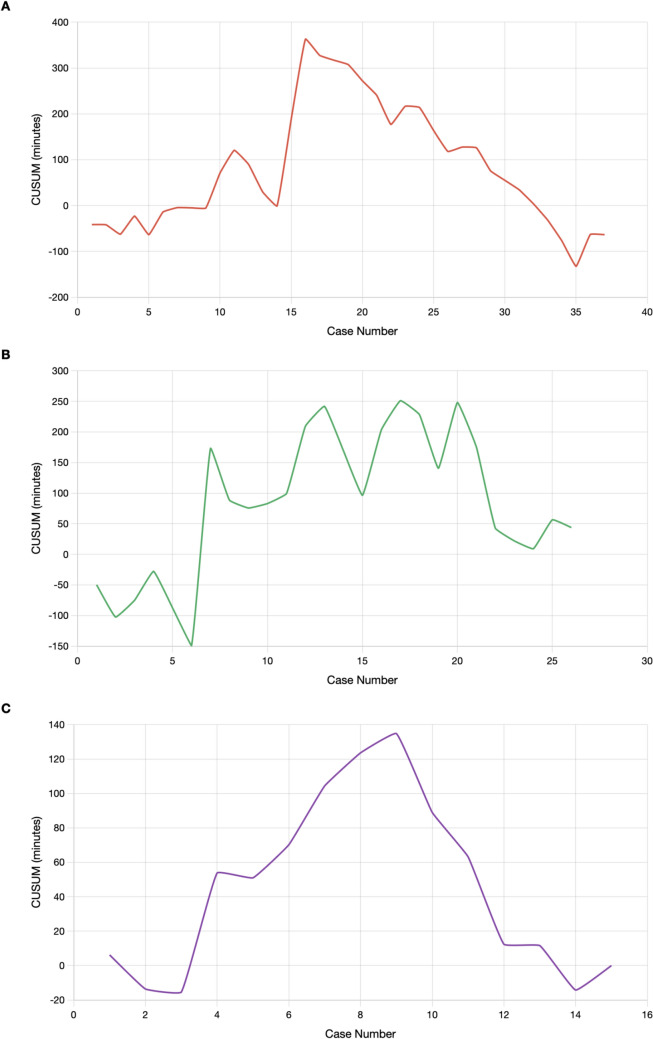
Procedure-specific cumulative sum (CUSUM) analyses showing learning curves for the three most common procedures. **a** Right hemicolectomy (*n* = 37): non-monotonic pattern with initial efficiency below the procedure mean (170 min), mid-series variation due to case complexity, and stabilization at approximately case 14. **b** Anterior rectal resection (*n* = 26): classic learning curve pattern showing initial operative times above the procedure mean (253 min), with progressive improvement and stabilization at case 11, achieving a 77-min reduction between early and late phases. **c** Sigmoidectomy (*n* = 15): rapid learning curve with stabilization at case 9, demonstrating 28-min improvement from initial to final phase. Note: *Y*-axis scales differ between graphs to optimize visualization of each procedure’s specific pattern

Procedure-specific CUSUM analyses demonstrated distinct patterns (Fig. 3).

#### Right hemicolectomy

Non-monotonic pattern with initial efficiency, mid-series variation, and late stabilization at approximately 14 cases (Table 2). Temporal analysis showed operative times of 178 min (first 12 cases), 168 min (cases 13–24), and 194 min (last 13 cases), with the late increase attributed to two complex outlier cases.

**Table 2:** Outcomes by malignancy status

Parameter	Benign (*n* = 22)	Malignant (*n* = 78)	*p* value
Length of stay (days)	5.0 (4.0–7.0)	7.0 (5.0–11.0)	0.042*
Overall complications	6 (27.3%)	22 (28.2%)	0.932
Severe complications (CD ≥ III)	3 (13.6%)	6 (7.7%)	0.412
Operative time (min)	165 (140–210)	185 (150–245)	0.138
Re-intervention	1 (4.5%)	2 (2.6%)	0.524

Data presented as *n* (%) or median (IQR)

*Statistically significant

*CD* Clavien–Dindo

#### Anterior rectal resection

Classic learning curve with progressive improvement from 261 min (first 9 cases) to 184 min (last 8 cases), representing a 77-min reduction despite one major outlier (575 min) (Table 3).

**Table 3:** Outcomes by procedure type

Procedure	*N*	Operative time (min)	Overall complications	Severe complications (CD ≥ III)	Length of stay (days)
Right hemicolectomy	37	180 (150–220)	9 (24.3%)	3 (8.1%)	5.0 (4.0–7.0)
Anterior rectal resection	26	240 (200–300)	9 (34.6%)	1 (3.8%)	8.0 (6.0–12.0)
Sigmoidectomy	15	150 (120–180)	2 (13.3%)	0 (0.0%)	5.0 (4.0–6.0)
Left hemicolectomy	6	195 (165–245)	4 (66.7%)	1 (16.7%)	12.5 (7.0–16.0)
Rectopexy	3	135 (120–150)	0 (0.0%)	0 (0.0%)	4.0 (3.0–5.0)
Abdominoperineal resection	3	285 (260–310)	1 (33.3%)	1 (33.3%)	10.0 (8.0–14.0)
Other procedures	10	210 (165–255)	3 (30.0%)	1 (10.0%)	7.0 (5.0–10.0)

Data presented as median (IQR) or *n* (%)

Other procedures include total colectomy, ileocecal resection, total proctectomy with IPAA, and ileocolic resection

*CD* Clavien–Dindo

#### Sigmoidectomy

Rapid stabilization at approximately 9 cases, with improvement from 161 min (first 7 cases) to 133 min (last 8 cases).

## Discussion

Our findings contribute novel insights into the evolving landscape of robotic colorectal surgery, particularly regarding the Hugo™ RAS platform’s performance in a real-world, early-adoption setting (Table 4). The deliberate pedagogical strategy of initiating our program with ten consecutive right hemicolectomies proved instrumental in achieving a 0% conversion rate throughout the entire series. This contrasts starkly with laparoscopic conversion rates reported in large trials, where the ROLARR trial documented a 7.4% conversion rate for laparoscopy versus 2.1% for robotics [2], and aligns with meta-analyses highlighting robotic surgery’s superiority in preserving minimally invasive approaches for complex cases [6, 7]. Similarly, laparoscopic series for low rectal cancer report conversion rates as high as 15–20% in anatomically challenging cases [8], whereas our robotic series eliminated this risk entirely. This underscores the platform’s ability to overcome anatomical challenges, such as narrow male pelvises or bulky tumors, which traditionally increase laparoscopic conversion risks [9].

**Table 4:** Temporal analysis: first 50 vs last 50 cases

Parameter	First 50 cases	Last 50 cases	*p* value
Demographics			
Age (years)	67.0 (58.0–76.0)	69.0 (60.0–79.0)	0.342
Male gender	26 (52.0%)	25 (50.0%)	0.841
ASA ≥ 3	18 (36.0%)	20 (40.0%)	0.683
Operative outcomes			
Operative time (min)	175 (145–235)	185 (150–245)	0.426
Conversion	0 (0.0%)	0 (0.0%)	1.000
Complications			
Overall morbidity	17 (34.0%)	11 (22.0%)	0.181
Clavien–Dindo I	3 (6.0%)	2 (4.0%)	0.617
Clavien–Dindo II	10 (20.0%)	4 (8.0%)	0.082
Clavien–Dindo IIIa	1 (2.0%)	3 (6.0%)	0.617
Clavien–Dindo IIIb	3 (6.0%)	2 (4.0%)	1.000
Major complications (≥ III)	4 (8.0%)	5 (10.0%)	1.000
Recovery			
Length of stay (days)	6.0 (5.0–10.0)	6.0 (5.0–10.0)	0.872
Re-intervention	2 (4.0%)	1 (2.0%)	1.000

Data presented as *n* (%) or median (IQR)

The global CUSUM analysis revealed a learning curve that stabilized after approximately 50 cases, with the initial downward deviation reflecting our strategic choice to begin with right hemicolectomies. This approach allowed systematic skill acquisition with the Hugo™ RAS platform before advancing to more complex pelvic procedures. Notably, our median operative time (180 min) compares favorably to early da Vinci colorectal series (210–240 min) [10, 11] and laparoscopic times for comparable procedures (195–220 min) [12], suggesting that the Hugo™ RAS platform allows for efficient operative workflows even during the early adoption phase.

### Procedure-specific learning curves

Our procedure-specific CUSUM analyses (Fig. 3) revealed distinct learning patterns for different operations. Right hemicolectomy (Fig. 3a) showed a non-monotonic pattern with initial efficiency during the first 12 cases (mean 178 min), improvement in the middle phase (168 min), followed by an increase in the final third (194 min). This late increase was influenced by two complex outlier cases requiring extended operative times, representing more challenging anatomical or pathological scenarios encountered.

Anterior rectal resection (Fig. 3b) demonstrated the most dramatic improvement, with operative times decreasing from 261 min in the first third to 184 min in the final third—a 77-min reduction. This substantial improvement, despite including one major outlier (575 min), reflects the team’s progressive mastery of pelvic dissection with the robotic platform. Similarly, sigmoidectomy (Fig. 3c) showed consistent improvement from 161 to 133 min (28-min reduction), with rapid stabilization after approximately 9 cases.

### Morbidity and complications

The 28% overall morbidity rate and 5% major complication rate (Clavien–Dindo ≥ IIIb) align with robotic colorectal literature [6, 13]. The temporal comparison between the first and last 50 cases demonstrated a reduction in overall complications from 34% to 22%, primarily driven by fewer minor complications, while major complication rates remained low and stable. This pattern suggests technical refinement over time while maintaining consistent safety standards throughout the learning process.

The 8% incidence of intra-abdominal abscesses, while higher than mature robotic platforms (3–5%) [2, 5], aligns with laparoscopic rectal surgery benchmarks (5–10%) [14, 15] and showed temporal improvement. This likely reflects initial learning with energy device integration and was addressed through optimized port placement and standardized dissection techniques. Importantly, our anastomotic leak rate (3%) matches outcomes from high-volume da Vinci centers (2–4%) [2], underscoring Hugo™ RAS’s reliability in critical tasks.

### Gender differences and risk factors

The significant association between male gender and complications (*p* = 0.011), with male patients experiencing a 39.2% complication rate versus 16.3% in female patients, warrants further investigation. While prior studies note higher postoperative morbidity in male patients after rectal surgery [9], this marked difference in our robotic series may reflect anatomical factors such as narrower pelvises complicating dissection, or biological differences in tissue healing [16]. This finding underscores the need for sex-specific risk stratification in future robotic colorectal studies.

### Oncologic outcomes

Oncologic outcomes, including a 93% R0 resection rate and median lymph node harvest of 20, align with benchmarks for robotic TME and colectomies [6, 17]. The lymph node yield surpasses both the minimum of 12 nodes recommended by AJCC guidelines [18] and typical laparoscopic averages of 15–18 nodes [19], aligning with high-volume robotic centers using the da Vinci system, which report harvests of 18–22 nodes [20]. Recent studies, such as a 2024 meta-analysis, further support that robotic surgery achieves higher lymph node yields compared to laparoscopic approaches, with a statistically significant difference (*p* = 0.04) [21]. Additionally, a 2019 systematic review found robotic right hemicolectomy to yield more nodes in two studies (*p* < 0.05) [22], reinforcing our results’ alignment with robotic outcomes. The CLASICC trial reported a median of 12 nodes for laparoscopic colectomies [23], while robotic superiority in lymph node retrieval is often attributed to precise mesocolic dissection [24]. Our R0 resection rate of 93% reflects excellent outcomes, consistent with recent evidence showing no significant difference between robotic and laparoscopic approaches, both achieving rates above 90% in specialized centers (OR 0.99, *p* = 0.98) [25]. Historically, robotic benchmarks suggested 90–95% [26], while laparoscopic rates were around 85–90% [23], but current data indicate equivalence. In rectal cancer cases, the quality of total mesorectal excision (TME) with robotic assistance is well documented [27], contributing to our high R0 rate.

The median hospital stay of 6 days (IQR 5–10) aligns closely with established robotic colorectal series and surpasses many laparoscopic benchmarks. High-volume da Vinci studies report median stays of 5–7 days for colectomies and 6–8 days for rectal resections [5, 28], while laparoscopic series often document longer stays (7–9 days) even in enhanced recovery pathways [8, 12]. The COLOR II trial reported a median stay of 8 days [8], whereas robotic trials like ROLARR noted 7 days [2]. Our shorter stay may reflect reduced postoperative pain and ileus rates inherent to robotic precision. Despite our 28% morbidity rate, this advantage persists, which mirrors laparoscopic complication rates (25–35%) [29]. Notably, laparoscopic conversions—a key driver of prolonged hospitalization—were absent in our cohort. In contrast, converted laparoscopic cases in the CLASICC trial had stays exceeding 12 days [23], highlighting robotics’ role in avoiding open surgery’s recovery burdens.

### Technical adaptations and platform-specific considerations

Hugo™ RAS’s modular architecture offered logistical advantages, particularly for multiquadrant procedures. However, early cases required iterative refinement of port placement to minimize arm collisions, leading to adoption of a “staggered” configuration. For splenic flexure mobilization during low anterior resections in selected patients, we opted for a technique using the fourth robotic arm as the main right hand of the surgeon and working only with three arms, avoiding the need for re-docking while maintaining adequate exposure. These platform-specific adaptations, combined with standardized troubleshooting protocols, contributed to the absence of unplanned re-docking and zero conversions throughout the series. While the system’s disposable instruments and reusable trocars may reduce costs compared to da Vinci, our study did not capture economic data—a critical gap given ongoing debates about robotic surgery’s cost-effectiveness [30].

### Learning curve and CUSUM analysis

The global CUSUM plot demonstrated an initial downward deviation, reflecting the deliberate choice to begin with 10 consecutive right hemicolectomies before progressing to more complex procedures, including pelvic and multiquadrant resections. Operative performance stabilized after approximately 50 cases, indicating improved efficiency over time [11]. This plateau reflects a progressive convergence of operative durations toward the mean, consistent with enhanced team coordination and increased procedural familiarity [31]. These findings highlight the value of structured training programs to minimize early variability and support the need for multicenter studies with larger cohorts to refine the learning curve and optimize the integration of the Hugo™ RAS platform into colorectal surgical practice [32, 33].

In addition to the global CUSUM analysis, procedure-specific CUSUM curves were generated for right hemicolectomy, anterior rectal resection, and sigmoidectomy (Fig. 3). These curves demonstrated the expected early variability followed by plateauing within each procedure type, occurring at approximately 14 cases for right hemicolectomy, 11 cases for anterior rectal resection, and 9 cases for sigmoidectomy. Importantly, these procedure-specific plateaus were accompanied by a reduction in overall complication rates in the main series, primarily due to fewer low-grade complications, while severe complication rates remained low and stable throughout the learning curve. This suggests that efficiency gains achieved within individual procedure types translated into tangible perioperative benefits, supporting the relevance of procedure-specific learning in addition to the global case mix.

### Economic implications

Although direct cost data were not collected, several clinical outcomes have important economic implications. In our cohort, the observed absence of conversions eliminates costs associated with prolonged operative time, extended hospitalization, and open surgery morbidity. The numerical decline in overall complications and the stabilization of operative times are established cost drivers that tend to shorten length of stay and reduce readmissions. These factors suggest that Hugo™ RAS could narrow the economic gap between robotic and laparoscopic approaches, particularly as teams achieve proficiency.

### Why our results matter in the robotic-laparoscopic debate

The shorter hospital stay and 0% conversion rate position Hugo™ RAS as a bridge between laparoscopic accessibility and robotic precision. While laparoscopy remains cost-effective for straightforward cases [12], robotics’ superiority in complex pelvic surgery is undeniable. Laparoscopic low rectal resections have conversion rates up to 15% in male patients with narrow pelvises [8], whereas our robotic approach eliminated conversions entirely. This aligns with meta-analyses showing robotics reduces conversion risk by 60% compared to laparoscopy [34].

Economically, although robotic surgery is often criticized for higher upfront costs, the absence of conversions and shorter hospital stays may offset expenses. Research indicates that robotic procedures may incur costs approximately 15% higher than laparoscopic surgeries because of advanced technology and equipment expenses. Yet, they can reduce postoperative complications and readmissions, potentially lowering overall treatment costs [35]. Notably, a 2022 analysis of colon resection demonstrated that robotic surgery could be more cost-effective in high-volume centers, driven by shorter recovery times and fewer conversions [36]. These findings underscore the importance of institutional volume and patient selection in realizing the economic advantages of robotic surgery, though further research is needed to generalize these outcomes across diverse healthcare settings.

### Limitations and future directions

This study has limitations inherent to single-center, retrospective designs, including selection bias and short follow-up. The absence of a contemporaneous laparoscopic or da Vinci control group precludes direct comparative conclusions, though our outcomes align with published robotic benchmarks. The heterogeneous case mix, while reflecting real-world implementation, complicates subgroup analyses. All procedures were performed by two surgeons who concentrated their practice exclusively on Hugo™ RAS, potentially creating a learning bias not replicable in centers where surgeons maintain mixed robotic/laparoscopic practices.

Future research should prioritize multi-institutional collaborations to validate Hugo™ RAS’s efficacy across diverse practice settings. Long-term oncologic outcomes, including local recurrence and survival, are essential to confirm its non-inferiority to established platforms. Cost–benefit analyses and ergonomic studies comparing surgeon fatigue across systems would further inform adoption decisions.

## Conclusions

In this early institutional experience with 100 consecutive cases, the Hugo™ RAS platform proved to be a safe and effective tool for colorectal surgery, achieving zero conversions, oncologic metrics within benchmark standards, and morbidity rates comparable to established robotic systems.

Our strategic approach of initiating the program with standardized right hemicolectomies facilitated systematic learning before progressing to complex pelvic procedures. Global and procedure-specific CUSUM analyses demonstrated that operative proficiency was attained after approximately 50 cases overall, with earlier plateaus for individual procedures.

The temporal numerical reduction in overall complications from 34% to 22%, primarily through fewer minor events while maintaining consistently low major morbidity rates, demonstrates the platform’s safety throughout the learning process.

These results support Hugo™ RAS as a valuable addition to minimally invasive colorectal surgery, providing procedural benchmarks that can guide other centers in structuring their adoption and training programs. The combination of zero conversions, improving complication profiles, and stabilizing operative times suggests the platform’s potential to broaden access to robotic surgery while maintaining high standards of care.

## Data Availability

The datasets analyzed during the current study are available from the corresponding author on reasonable request.

## References

[1] Weber PA, Merola S, Wasielewski A et al (2002) Telerobotic-assisted laparoscopic right and sigmoid colectomies for benign disease. Dis Colon Rectum 45:1689–1694 12473897 10.1007/s10350-004-7261-2

[2] Jayne D, Pigazzi A, Marshall H et al (2017) Effect of robotic-assisted vs conventional laparoscopic surgery on risk of conversion to open laparotomy among patients undergoing resection for rectal cancer: the ROLARR randomized clinical trial. JAMA 318:1569–1580 29067426 10.1001/jama.2017.7219 PMC5818805

[3] Lanfranco AR, Castellanos AE, Desai JP et al (2004) Robotic surgery: a current perspective. Ann Surg 239:14–21 14685095 10.1097/01.sla.0000103020.19595.7d PMC1356187

[4] Abdel Raheem A, Sheikh A, Kim DK et al (2017) Da Vinci Xi and Si platforms have equivalent perioperative outcomes during robot-assisted partial nephrectomy: preliminary experience. J Robot Surg 11:53–61 27342870 10.1007/s11701-016-0612-x

[5] Kim MJ, Park SC, Park JW et al (2018) Robot-assisted versus laparoscopic surgery for rectal cancer: a phase II open label prospective randomized controlled trial. Ann Surg 267:243–251 28549014 10.1097/SLA.0000000000002321

[6] Feng Q, Yuan W, Li T et al (2022) Robotic versus laparoscopic surgery for middle and low rectal cancer (REAL): short-term outcomes of a multi randomised controlled trial. Lancet Gastroenterol Hepatol 7:991–1004 36087608 10.1016/S2468-1253(22)00248-5

[7] Trastulli S, Cirocchi R, Desiderio J et al (2015) Robotic versus laparoscopic approach in colonic resections for cancer and benign diseases: systematic review and meta-analysis. PLoS ONE 10:e0134062 26214845 10.1371/journal.pone.0134062 PMC4516360

[8] van der Pas MH, Haglind E, Cuesta MA et al (2013) Laparoscopic versus open surgery for rectal cancer (COLOR II): short-term outcomes of a randomised, phase 3 trial. Lancet Oncol 14:210–218 23395398 10.1016/S1470-2045(13)70016-0

[9] Akiyoshi T, Kuroyanagi H, Oya M et al (2009) Factors affecting the difficulty of laparoscopic total mesorectal excision with double stapling technique anastomosis for low rectal cancer. Surgery 146:483–489 19715805 10.1016/j.surg.2009.03.030

[10] Park EJ, Kim CW, Cho MS et al (2014) Is the learning curve of robotic low anterior resection shorter than laparoscopic low anterior resection for rectal cancer?: a comparative analysis of clinicopathologic outcomes between robotic and laparoscopic surgeries. Medicine (Baltimore) 93:e109 25437022 10.1097/MD.0000000000000109 PMC4616378

[11] Bokhari MB, Patel CB, Ramos-Valadez DI et al (2011) Learning curve for robotic-assisted laparoscopic colorectal surgery. Surg Endosc 25:855–860 20734081 10.1007/s00464-010-1281-x PMC3044842

[12] Veldkamp R, Gholghesaei M, Bonjer HJ et al (2004) Laparoscopic resection of colon cancer: consensus of the European Association of Endoscopic Surgery (EAES). Surg Endosc 18:1163–1185 15457376 10.1007/s00464-003-8253-3

[13] Spinoglio G, Bianchi PP, Marano A et al (2018) Robotic versus laparoscopic right colectomy with complete mesocolic excision for the treatment of colon cancer: perioperative outcomes and 5-year survival in a consecutive series of 202 patients. Ann Surg Oncol 25:3580–3586 30218248 10.1245/s10434-018-6752-7

[14] Bonjer HJ, Deijen CL, Abis GA et al (2015) A randomized trial of laparoscopic versus open surgery for rectal cancer. N Engl J Med 372:1324–1332 25830422 10.1056/NEJMoa1414882

[15] Jeong S-Y, Park JW, Nam BH et al (2014) Open versus laparoscopic surgery for mid-rectal or low-rectal cancer after neoadjuvant chemoradiotherapy (COREAN trial): survival outcomes of an open-label, non-inferiority, randomised controlled trial. Lancet Oncol 15:767–774 24837215 10.1016/S1470-2045(14)70205-0

[16] Wilkinson NM, Chen H-C, Lechner MG et al (2022) Sex differences in immunity. Annu Rev Immunol 40:75–94 34985929 10.1146/annurev-immunol-101320-125133 PMC9805670

[17] Xiong B, Ma L, Huang W et al (2015) Robotic versus laparoscopic total mesorectal excision for rectal cancer: a meta-analysis of eight studies. J Gastrointest Surg 19:516–526 25394387 10.1007/s11605-014-2697-8

[18] Edge SB, Compton CC (2010) The American Joint Committee on Cancer: the 7th edition of the AJCC cancer staging manual and the future of TNM. Ann Surg Oncol 17:1471–1474 20180029 10.1245/s10434-010-0985-4

[19] Chang GJ, Rodriguez-Bigas MA, Skibber JM et al (2007) Lymph node evaluation and survival after curative resection of colon cancer: systematic review. J Natl Cancer Inst 99:433–441 17374833 10.1093/jnci/djk092

[20] Widmar M, Keskin M, Strombom P et al (2017) Lymph node yield in right colectomy for cancer: a comparison of open, laparoscopic and robotic approaches. Colorectal Dis 19:888–894 28649796 10.1111/codi.13786 PMC5642033

[21] Negrut RL, Cote A, Caus VA et al (2024) Systematic review and meta-analysis of laparoscopic versus robotic-assisted surgery for colon cancer: efficacy, safety, and outcomes-a focus on studies from 2020–2024. Cancers (Basel) 16:1552 38672635 10.3390/cancers16081552 PMC11048614

[22] Waters PS, Cheung FP, Peacock O et al (2020) Successful patient-oriented surgical outcomes in robotic vs laparoscopic right hemicolectomy for cancer—a systematic review. Colorectal Dis 22:488–499 31400185 10.1111/codi.14822

[23] Guillou PJ, Quirke P, Thorpe H et al (2005) Short-term endpoints of conventional versus laparoscopic-assisted surgery in patients with colorectal cancer (MRC CLASICC trial): multicentre, randomised controlled trial. Lancet 365:1718–1726 15894098 10.1016/S0140-6736(05)66545-2

[24] Protyniak B, Jorden J, Farmer R (2018) Multiquadrant robotic colorectal surgery: the da Vinci Xi vs Si comparison. J Robot Surg 12:67–74 28275893 10.1007/s11701-017-0689-x

[25] Khajeh E, Aminizadeh E, Dooghaie Moghadam A et al (2023) Outcomes of robot-assisted surgery in rectal cancer compared with open and laparoscopic surgery. Cancers (Basel) 15:839 36765797 10.3390/cancers15030839 PMC9913667

[26] Kang J, Yoon KJ, Min BS et al (2013) The impact of robotic surgery for mid and low rectal cancer: a case-matched analysis of a 3-arm comparison–open, laparoscopic, and robotic surgery. Ann Surg 257:95–101 23059496 10.1097/SLA.0b013e3182686bbd

[27] Pigazzi A, Luca F, Patriti A et al (2010) Multicentric study on robotic tumor-specific mesorectal excision for the treatment of rectal cancer. Ann Surg Oncol 17:1614–1620 20087780 10.1245/s10434-010-0909-3

[28] Park JS, Choi G-S, Park SY et al (2012) Randomized clinical trial of robot-assisted versus standard laparoscopic right colectomy. Br J Surg 99:1219–1226 22864881 10.1002/bjs.8841

[29] Schwenk W, Haase O, Neudecker J et al (2005) Short term benefits for laparoscopic colorectal resection. Cochrane Database Syst Rev 2005:CD003145 16034888 10.1002/14651858.CD003145.pub2 PMC8693724

[30] Sadri H, Fung-Kee-Fung M, Shayegan B et al (2023) A systematic review of full economic evaluations of robotic-assisted surgery in thoracic and abdominopelvic procedures. J Robot Surg 17:2671–2685 37843673 10.1007/s11701-023-01731-7 PMC10678817

[31] Panico G, Mastrovito S, Campagna G et al (2023) Robotic docking time with the Hugo™ RAS system in gynecologic surgery: a procedure independent learning curve using the cumulative summation analysis (CUSUM). J Robot Surg 17:2547–2554 37542580 10.1007/s11701-023-01693-w PMC10492716

[32] Romero-Marcos J-M, Sampson-Dávila J-G, Cuenca-Gómez C et al (2024) Colorectal procedures with the novel Hugo™ RAS system: training process and case series report from a non-robotic surgical team. Surg Endosc 38:2160–2168 38448626 10.1007/s00464-024-10760-8

[33] Rottoli M, Cardelli S, Calini G et al (2024) Outcomes of robotic surgery for inflammatory bowel disease using the Medtronic Hugo™ robotic-assisted surgical platform: a single center experience. Int J Colorectal Dis 39:158 39384631 10.1007/s00384-024-04736-2 PMC11464579

[34] Trastulli S, Farinella E, Cirocchi R et al (2012) Robotic resection compared with laparoscopic rectal resection for cancer: systematic review and meta-analysis of short-term outcome. Colorectal Dis 14:e134-156 22151033 10.1111/j.1463-1318.2011.02907.x

[35] Gorgun E, Cengiz TB, Ozgur I et al (2022) Outcomes and cost analysis of robotic versus laparoscopic abdominoperineal resection for rectal cancer: a case-matched study. Dis Colon Rectum 65:1279–1285 35195554 10.1097/DCR.0000000000002394

[36] Hancock KJ, Klimberg VS, Nunez-Lopez O et al (2022) Optimizing outcomes in colorectal surgery: cost and clinical analysis of robotic versus laparoscopic approaches to colon resection. J Robot Surg 16:107–112 33634355 10.1007/s11701-021-01205-8 PMC8384955

